# Retraction: Tanshinone IIA prevents left ventricular remodelling via the TLR4/MyD88/NF‐κB signalling pathway in rats with myocardial infarction. Dong‐Mei Wu, Yong‐Jian Wang, Xin‐Rui Han, Xin Wen, Lei Li, Lan Xu, Jun Lu and Yuan‐Lin Zheng. J Cell Mol Med. 2018; 22: 3058–3072 (https://doi.org/10.1111/jcmm.13557).

**DOI:** 10.1111/jcmm.16032

**Published:** 2021-01-04

The above article from Journal of Cellular and Molecular Medicine, published online on 10 March 2018 on Wiley Online Library (wileyonlinelibrary.com) in Volume 22, pp. 3058–3072, has been retracted by agreement between the authors, the journal Editor‐in‐Chief and John Wiley & Sons Ltd. The retraction has been agreed following an investigation based on allegations raised by a third party. The authors asked to retract this manuscript as raw experimental data were not accessible anymore and thus the validity of the overall results could not be confirmed.

